# Critical Role of CD2 Co-stimulation in Adaptive Natural Killer Cell Responses Revealed in NKG2C-Deficient Humans

**DOI:** 10.1016/j.celrep.2016.04.005

**Published:** 2016-04-21

**Authors:** Lisa L. Liu, Johannes Landskron, Eivind H. Ask, Monika Enqvist, Ebba Sohlberg, James A. Traherne, Quirin Hammer, Jodie P. Goodridge, Stella Larsson, Jyothi Jayaraman, Vincent Y.S. Oei, Marie Schaffer, Kjetil Taskén, Hans-Gustaf Ljunggren, Chiara Romagnani, John Trowsdale, Karl-Johan Malmberg, Vivien Béziat

**Affiliations:** 1 Center for Infectious Medicine, Department of Medicine Huddinge, Karolinska Institutet, 14186 Stockholm, Sweden; 2 The Biotechnology Centre of Oslo, University of Oslo, 0349 Oslo, Norway; 3 The KG Jebsen Center for Cancer Immunotherapy, Institute of Clinical Medicine, University of Oslo, 0318 Oslo, Norway; 4 Department of Cancer Immunology, Institute for Cancer Research, Oslo University Hospital, 0310 Oslo, Norway; 5 Cambridge Institute for Medical Research and Department of Pathology, Cambridge University, Cambridge CB2 0XY, UK; 6 Innate Immunity, Deutsches Rheuma-Forschungszentrum - A Leibniz Institute, 10117 Berlin, Germany; 7 Clinical Immunology and Transfusion Medicine, Department for Laboratory Medicine, Karolinska Institute, 17177 Stockholm, Sweden; 8 Centre for Molecular Medicine Norway, Nordic EMBL Partnership, University of Oslo and Oslo University Hospital, 0318 Oslo, Norway; 9 K.G. Jebsen Inflammation Research Centre, University of Oslo, 0318 Oslo, Norway; 10 Department of Infectious Diseases, Oslo University Hospital, 0424 Oslo, Norway; 11 Laboratory of Human Genetics of Infectious Diseases, Necker Branch, INSERM U1163, 75015 Paris, France; 12 University Paris Descartes, Imagine Institute, 75270 Paris, France

## Abstract

Infection by human cytomegalovirus (HCMV) leads to NKG2C-driven expansion of adaptive natural killer (NK) cells, contributing to host defense. However, approximately 4% of all humans carry a homozygous deletion of the gene that encodes NKG2C (*NKG2C*^−/−^). Assessment of NK cell repertoires in 60 *NKG2C*^−/−^ donors revealed a broad range of NK cell populations displaying characteristic footprints of adaptive NK cells, including a terminally differentiated phenotype, functional reprogramming, and epigenetic remodeling of the interferon (IFN)-γ promoter. We found that both NKG2C^−^ and NKG2C^+^ adaptive NK cells expressed high levels of CD2, which synergistically enhanced ERK and S6RP phosphorylation following CD16 ligation. Notably, CD2 co-stimulation was critical for the ability of adaptive NK cells to respond to antibody-coated target cells. These results reveal an unexpected redundancy in the human NK cell response to HCMV and suggest that CD2 provides “signal 2” in antibody-driven adaptive NK cell responses.

## Highlights

• NKG2C^−/−^ donors have normal T cell immunity to cytomegalovirus
• NKG2C^−/−^ donors have normal frequencies of adaptive NK cells
• CD2 is critical for antibody-triggered responses by adaptive NK cells
• CD2 synergizes with NKG2C in classical adaptive NK cells

## Graphical Abstract

**Figure:**
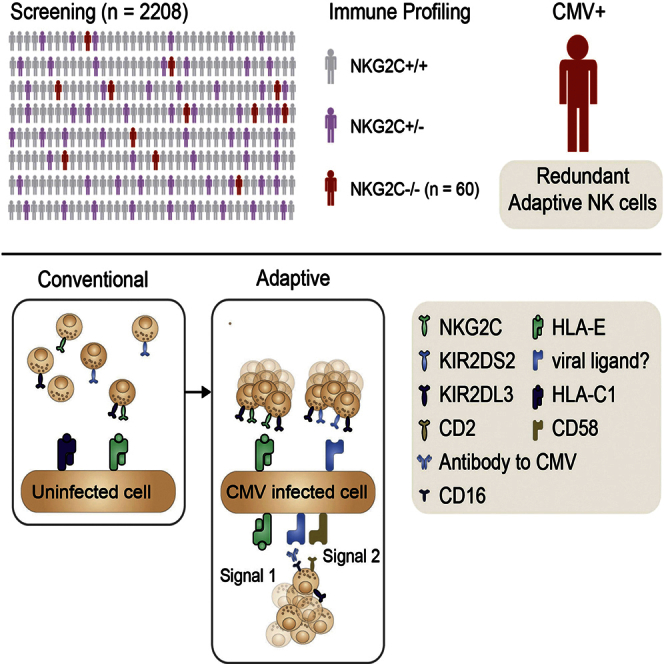

## Teaser

Liu et al. demonstrate the emergence of redundant adaptive NK cell subsets in NKG2C^−/−^ donors. Functional studies unraveled a critical role for CD2 in antibody-dependent responses by adaptive NK cells, paving the way for new strategies to harness their cytotoxic potential in cell therapy.

## Introduction

Human cytomegalovirus (HCMV) is a persistent betaherpes virus with a worldwide prevalence ranging between 50% and 100% of the population depending on socioeconomic factors. Congenital HCMV infection is a leading cause of sensorineural hearing loss in children and a significant cause of neurodevelopmental delay (Manicklal et al., 2013). Immunocompromised patients with AIDS, severe combined immunodeficiency (SCID), or those having received immunosuppressive treatment in conjunction with hematopoietic stem cell transplantation (HSCT), frequently experience life-threatening HCMV infection. Adaptive immunity plays a crucial role in the control of HCMV (Crough and Khanna, 2009). HCMV-specific T cells have a terminally differentiated phenotype and can represent up to 40% of the total T cell memory pool (Sylwester et al., 2005, van Lier et al., 2003).

Natural killer (NK) cells are innate lymphocytes involved in numerous physiological processes including reproduction (Parham and Moffett, 2013) and immunity to infections (Jost and Altfeld, 2013). Recent advances in NK cell biology suggest that NK cells display adaptive features during CMV infection, contributing to viral control (Vivier et al., 2011). Unlike T and B cell immune responses, CMV-driven adaptive NK cell responses do not rely on receptor rearrangement. In mice, infection with mouse CMV (MCMV) leads to a clonal expansion of NK cells expressing the activating receptor Ly49H, which binds to the MCMV-encoded protein m157 (Arase et al., 2002). Optimal differentiation of adaptive Ly49H^+^ NK cells depends on delivery of “signal 2,” provided through co-stimulation via DNAM-1 (Nabekura et al., 2014). The expansion and contraction within the Ly49H^+^ NK cell population result in a pool of memory NK cells that mediate long-lasting protection against subsequent challenges with the virus (Sun et al., 2009).

In humans, the NK cell response against HCMV results in a stable imprint of highly differentiated NK cells expressing DAP12-coupled receptors including NKG2C and activating killer cell immunoglobulin-like receptors (KIRs) (Béziat et al., 2013, Gumá et al., 2004). Expansion of these NK cell subsets, referred to as adaptive NK cells, have been observed following HCMV reactivation in transplanted patients (Della Chiesa et al., 2014, Foley et al., 2012, Lopez-Vergès et al., 2011) and was associated with viral control in a T^–^B^+^NK^+^ SCID patient experiencing acute primary HCMV infection (Kuijpers et al., 2008).

Despite accumulating evidence that NKG2C plays a central role in the NK cell response against HCMV, NKG2C is dispensable for survival and reproduction. Approximately 20% of human haplotypes carry a full deletion of the *KLRC2*/*NKG2C* gene (hereafter referred to as *NKG2C* only) (Miyashita et al., 2004, Moraru et al., 2012b, Thomas et al., 2012). Accordingly, approximately 4% of the human population completely lacks the *NKG2C* gene (*NKG2C*^−/−^). Although lack of *NKG2C* has been linked to an increased risk of HIV progression (Thomas et al., 2012), it is not overrepresented in children with severe congenital HCMV infection (Noyola et al., 2012), and HCMV seropositive *NKG2C*^−/−^ adults remain healthy without specific symptoms. The lack of a clinical phenotype raises the question of whether adaptive NK cell responses are physiologically relevant or if other cellular mechanisms compensate for the loss of NKG2C-driven adaptive NK cell responses. Here, comparative studies of HCMV immunity in large cohorts of NKG2C-sufficient and -deficient individuals revealed an unexpected redundancy in the adaptive NK cell response and point to a critical role for CD2 in providing co-stimulation for NKG2C- and antibody-mediated triggering of adaptive NK cells.

## Results

### Minimal Imprint in T Cell Immunity in Individuals Carrying Homozygous Deletion of NKG2C

The absence of more severe HCMV infection in *NKG2C*^−/−^ donors indicates the existence of redundant pathways for the control of the infection. To enable a comprehensive analysis of the immune system in *NKG2C*^−/−^ donors, we screened 2,208 healthy blood donors and identified 81 *NKG2C*^−/−^ donors, corresponding to a frequency of 3.7% in the Swedish population, in line with frequencies reported in other populations (Miyashita et al., 2004, Moraru et al., 2012b, Thomas et al., 2012). We then prospectively obtained buffy coats from 60 of these for downstream analyses. The demographics of the *NKG2C*^−/−^ cohort and of the age-matched *NKG2C*^+^ controls (*NKG2C*^+/−^ and NKG2C^+/+^) are summarized in Table S1.

**Figure 1:**
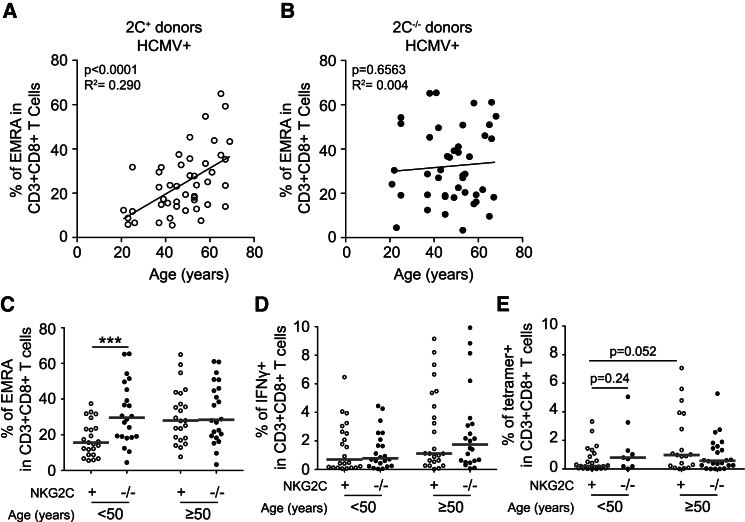
Homozygous *NKG2C* Deletion Is Associated with Accumulation of Terminally Differentiated Effector Memory CD45RA^+^ T Cells (A and B) Frequency of EMRA CD8 T cells in HCMV^+^*NKG2C*^+^ donors (A) and HCMV^+^*NKG2C*^−/−^ donors (B) plotted against the age of the donor. (C) Frequency of EMRA CD8 T cells in donors <50 and >50 years old as a function of *NKG2C* deletion. (D) Frequency of IFN-γ^+^ CD8 T cells after overnight stimulation with pp65 overlapping peptide pools. (E) Frequency of HCMV-specific CD8 T cells as defined by HLA-A^∗^02 or HLA-B^∗^07 tetramers refolded with pp65-derived peptides. Gray lines represent the median value within each group.

We analyzed the impact of homozygous *NKG2C* deletion on the differentiation profile and the anti-HCMV response of CD4 and CD8 T cells from NKG2C^−/−^ donors as compared to NKG2C^+^ (*NKG2C*^+/+^ or *NKG2C*^+/−^) donors (Figures 1, S1, and S2). We found that the *NKG2C* deletion resulted in a slight but statistically significant accumulation of terminally differentiated effector memory CD45RA^+^ (CCR7^–^CD45RA^+^) cells in the CD8^+^ T cell compartment (24.1 ± 14.4 versus 32.3 ± 16.9, p = 0.014), whereas no significant changes were observed for any of the other CD8 T cell subsets studied (Figures S1A and S1B). Interestingly, the accumulation of mature CD8 T cells was particularly visible in young and middle-age individuals (17.8 ± 9.6 versus 32.07 ± 17.2, p = 0.001; Figures 1A–1C). However, CD8 T cell responses following stimulation with overlapping peptide pools derived from the HCMV proteins IE-1, IE-2, and pp65 were identical in *NKG2C*^−/−^ and *NKG2C*^+^ individuals, regardless of age (Figures 1D, S1C, and S1D). These results were confirmed by using HLA-A^∗^02 and HLA-B^∗^07 tetramers refolded with pp65-derived immunodominant peptides to detect HCMV-specific CD8 T cells (Figures 1E, S1E, and S1F). Similarly, *NKG2C* deletion was not associated with any significant phenotypic or functional differences in CD4^+^ T cells (Figure S2) and did not imprint B cell differentiation (Figure S3). Thus, despite an accumulation of terminally differentiated CD8 T cells in young NKG2C^−/−^ individuals, our results show that no major reshaping of T and B cell immunity to HCMV takes place in NKG2C-deficient individuals.

### Adaptive NK Cell Response to HCMV in *NKG2C*^−/−^ Individuals

**Figure 2:**
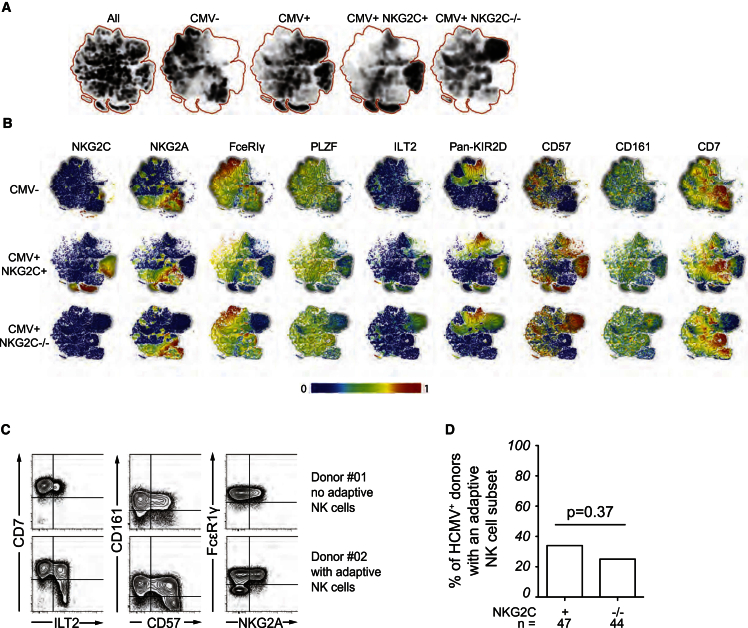
Redundant Adaptive NK Cell Response against HCMV in *NKG2C*^−/−^ Donors (A and B) Unbiased bulk analysis of five HCMV^–^, five HCMV^+^*NKG2C*^+/+^, and five HCMV^+^*NKG2C*^−/−^ donors using the t-SNE algorithm. (A) Density plots show the clustering of cell phenotypes in donors with and without adaptive NK cell populations stratified based on CMC seropositivity and presence/absence of the NKG2C gene. (B) Differentiation marker distribution in the clusters defined for HCMV^–^ (top), HCMV^+^NKG2C^+/+^ (middle), and HCMV^+^NKG2C^−/−^ donors (bottom). Color codes indicate the expression intensity from the lowest (blue) to the highest (red). (C) Representative fluorescence-activated cell sorting (FACS) plots of donors with (donor #02) and without (donor #01) evidence of NK cell adaptive response as revealed by the upregulation of CD57, LILRB1, and downregulation of CD7, CD161, and FcεR1γ. (D) Frequency of HCMV^+^*NKG2C*^+^ and HCMV^+^*NKG2C*^−/−^ donors with a NK cell expansion.

We recently reported that, among HCMV^+^ individuals, some displayed an expansion of NKG2C-negative NK cells, all of which expressed activating KIRs (Béziat et al., 2013). Such adaptive NK cells were identified by their highly differentiated phenotype, manifested by reduced expression of CD7, CD161, and FcεR1γ and higher expression of CD57 and LILRB1 (Béziat et al., 2013, Béziat et al., 2014, Zhang et al., 2013). To examine the possible existence of adaptive NK cells in *NKG2C*^−/−^ donors, we analyzed multi-parametric flow cytometry data by non-linear dimensionality reduction using t-distributed stochastic neighbor embedding (t-SNE) (Figure 2A) (Amir et al., 2013). The t-SNE algorithm clusters cells according to their expression of multiple parameters and visualizes high-dimensional data in two-dimensional representations, avoiding the bias introduced by manual gating of specific subsets. This analysis clearly revealed clusters of cells sharing the phenotypic hallmarks of adaptive NK cells in *NKG2C*^−/−^ donors (Figures 2A and 2B). In fact, NKG2C was the only marker that distinguished adaptive NK cell clusters in NKG2C-sufficient and -deficient donors. Applying stringent phenotypic criteria to assign a given cell population as adaptive, we quantified the frequency of *NKG2C*^+^ and *NKG2C*^−/−^ donors with imprints of adaptive NK cell responses (Figures 2C and 2D). Donors considered as carrier of an adaptive NK cell subset were defined on the basis of significant expansion of cells, representing >10% of CD56^dim^ NK cells, displaying at least four of the five phenotype characteristics (low CD7, low CD161, low FcεR1γ, high CD57, or high LILRB1). Among the 47 HCMV^+^NKG2C^+^ individuals in the control cohort, 16 (34%) had a population of adaptive NK cells, in agreement with the 38% reported previously (Béziat et al., 2013). Surprisingly, we found that 11 (25%) of the 44 HCMV^+^*NKG2C*^−/−^ donors had a significant population (>10%) of adaptive NK cells, a proportion not significantly different from the *NKG2C*^+^ individuals (Fisher’s exact t test: p = 0.27) (Figure 2D). Notably, none of the 14 *NKG2C*^+^ or 16 *NKG2C*^−/−^ HCMV seronegative donors harbored such expansions (data not shown), suggesting that, like *NKG2C*^+^ donors, the expansions observed in *NKG2C*^−/−^ individuals were related to HCMV infection.

### Adaptive NKG2C^−/−^ NK Cells Preferentially Express Self HLA-Specific KIR and Share Functional Attributes with NKG2C^+^ Adaptive NK Cells

**Figure 3:**
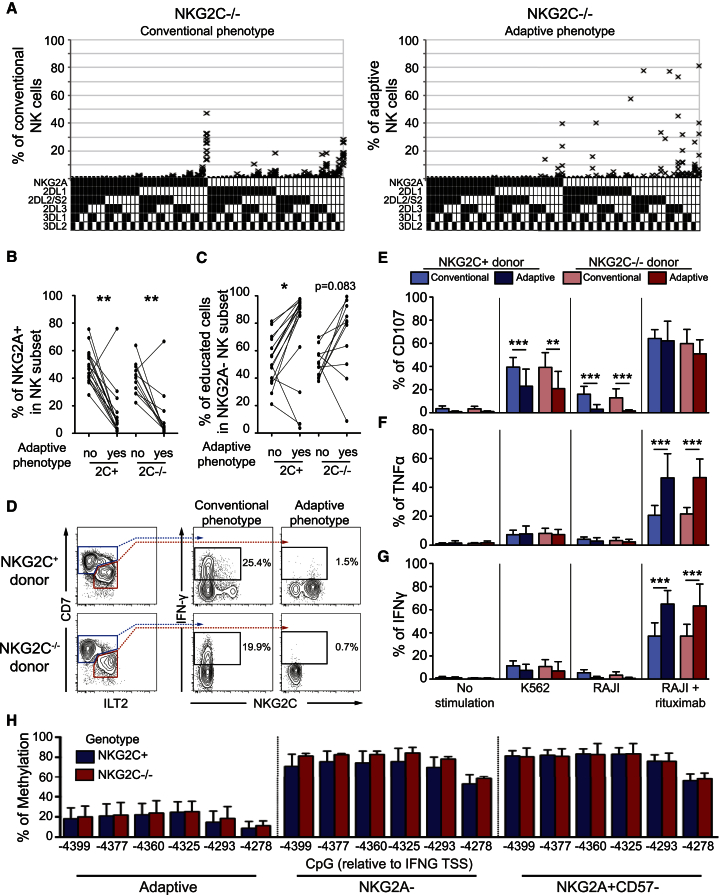
Adaptive NK Cells in *NKG2C*^−/−^ Donors Share Most Attributes of NKG2C-Expressing Adaptive NK Cells (A) NKG2A and KIR repertoire in conventional (left column) and adaptive (right column) NK cells of *NKG2C*^−/−^ donors. (B and C) Frequency of NKG2A^+^ NK cells (B) and educated cells (C) in adaptive and conventional NK cells of *NKG2C*^+^ and *NKG2C*^−/−^ donors. (D) Representative intracellular IFN-γ production by conventional and adaptive NK cells in NKG2C^+^ (n = 16) and NKG2C^−/−^ (n = 11) donors after overnight stimulation with IL-12 and IL-18. (E–G) Functional assay of conventional CD56^dim^ NK cells as compared to adaptive NK cells from HCMV^+^*NKG2C*^+^ (n = 16) or HCMV^+^*NKG2C*^−/−^ (n = 11) donors. Cell-surface expression of CD107a (E) and intracellular expression of TNF (F) and IFN-γ. (G) were assessed after 6 hr of stimulation with K562 target cells or RAJI target cells in the presence of anti-CD20 (rituximab, 1 μg/ml). (H) CpG methylation profile relative to transcriptional start site (TSS) of the IFN-γ promoter in adaptive NK cells compared to NKG2A^–^ and NKG2A^+^CD57^–^ subsets. Four NKG2C^−/−^ and six NKG2C^+/+^ donors were analyzed. The error bars represent the SEM.

To further characterize the adaptive NK cell population in *NKG2C*^−/−^ donors, we assessed their KIR repertoires. Strikingly, as in *NKG2C*^+^ donors (Béziat et al., 2012, Béziat et al., 2013), adaptive NK cell populations in *NKG2C*^−/−^ donors displayed profound deviations in their KIR repertoires (Figures 3A and S4) with preferential expression of self-KIR and low frequencies of NKG2A (Figures 3B and 3C). More importantly, they shared functional attributes of adaptive NK cells observed in *NKG2C*^+^ individuals. First, they had a poor ability to produce interferon (IFN)-γ after interleukin-12 (IL-12)/IL-18 stimulation (Figure 3D). Second, they displayed decreased degranulation capacity, measured via cell-surface expression of CD107a, upon direct interaction with K562 (p = 0.0046) and RAJI (p = 0.0005) cells and displayed enhanced IFN-γ (p < 0.0001) and tumor necrosis factor (TNF) (p = 0.0002) production against antibody-coated target cells compared to conventional NK cells (Figures 3E–3G). Third, in line with the enhanced ability to produce IFN-γ, compared to conventional NK cells, they displayed a clear epigenetic remodeling associated with demethylation of CpG motifs in the conserved noncoding sequence (CNS) 1 of the *IFNG* locus (Figure 3H), which was shown to be exclusively demethylated in NKG2C-expressing expansions from HCMV^+^ individuals (Luetke-Eversloh et al., 2014).

Altogether, these data demonstrate that *NKG2C*^−/−^ individuals develop HCMV-driven adaptive NK cell responses at similar frequencies and with similar epigenetic, phenotypic, and functional properties as *NKG2C*^+^ individuals.

### Adaptive Response to HCMV Occurs Independently of Activating KIRs in *NKG2C*^−/−^ Individuals

**Figure 4:**
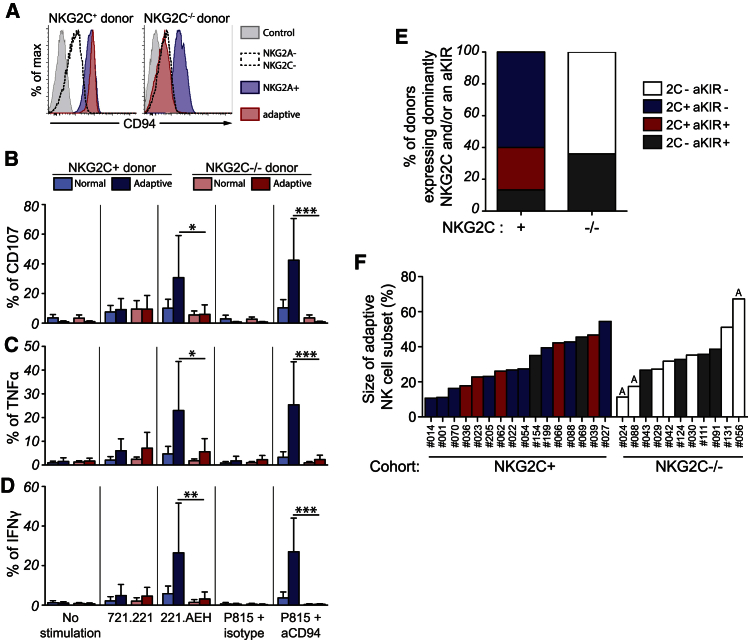
Adaptive Responses of NK Cells in NKG2C^−/−^ Individuals Independently of CD94 and Activating KIRs (A) CD94 expression by the NKG2A^+^ (blue lines), the NKG2A^–^NKG2C^–^ (black dotted lines), and the adaptive (red lines) subsets of HCMV^+^*NKG2C*^+^ (left) and HCMV^+^*NKG2C*^−/−^ (right) donors as compared to a fluorescence minus one (FMO) control. (B–D) Functional assay of conventional CD56^dim^ NK cells as compared to adaptive NK cells from HCMV^+^*NKG2C*^+^ (n = 16) or HCMV^+^*NKG2C*^−/−^ donors (n = 11). Cell-surface expression of CD107a (B) and intracellular expression of TNF (C) and IFN-γ (D) were assessed after 6 hr of stimulation with target cells expressing HLA-E (221.AEH) or not (721.221) or redirected stimulation with P815 and mouse anti human CD94 (10 μg/ml) or an isotype control. Error bars represent the SEM. (B–D) Adaptive subsets in NKG2C^+/+^ and NKG2C^−/−^ donors were defined by the FcεR1γ^–^CD57^+^ phenotype. (E) Frequency of *NKG2C*^+^ and *NKG2C*^−/−^ donors having the indicated phenotype as their dominating (>50%) adaptive subset. (F) Size of the adaptive NK cell subset in *NKG2C*^+^ and *NKG2C*^−/−^ donors. Three haplotype A/A donors are marked with an A at the top of their respective columns.

The identification of adaptive NK cells in donors lacking *NKG2C* raised the question of which potential activating receptors might contribute to the expansion of this subset. Among other genes, the NK gene complex on chromosome 12 encodes NKG2E, an activating receptor that also forms functional heterodimers with CD94 and recognizes HLA-E (Lanier et al., 1998, Lazetic et al., 1996). Since CD94 was at least weakly expressed on all NK cells in both *NKG2C*^+^ and *NKG2C*^−/−^ donors (Figure 4A), we asked whether an alternative activating CD94/NKG2 heterodimer was functional on adaptive NK cells in the absence of NKG2C. To this end, we stimulated NK cells with 221.AEH cells or with P815 target cells coated with anti-CD94 (Figures 4B–4D). These experiments demonstrated that neither triggering of CD94 (P815 + anti-CD94) nor stimulation with the natural HLA-E ligand (221.AEH) induced functional responses in adaptive NK cells from *NKG2C*^−/−^ donors when compared with *NKG2C*-expressing adaptive NK cells. These results exclude the involvement of NKG2E in the expansion of adaptive NK cell subsets in *NKG2C*^−/−^ donors.

Previous reports suggest that activating KIRs may compensate for the loss of NKG2C in donors with a homozygous *NKG2C* deletion (Béziat et al., 2013, Della Chiesa et al., 2014). Accordingly, we examined the relative contribution of NKG2C and activating KIRs to the adaptive NK cell pool in each donor (Figure 4E). In *NKG2C*^+^ donors, 60% of the expansions expressed only NKG2C, 27% co-expressed NKG2C and an activating KIR (KIR2DS1, KIR2DS2, KIR3DS1, or KIR2DS4), whereas 13% of the expansions expressed predominantly an activating KIR. Surprisingly, we found similar overall frequencies of activating KIR-positive expansions in *NKG2C*^−/−^ individuals (36%) (Figure 4E). Moreover, the magnitude of the adaptive response, determined as the fraction of adaptive NK cells within the total NK cell subset, did not differ in donors with and without the *NKG2C* deletion and seemed to be independent of the activating receptor composition (Figure 4F). Although our phenotypic analysis did not include KIR2DS3 and KIR2DS5, the detection of three haplotype A/A donors among the 11 *NKG2C*^−/−^ donors with an expansion allowed us to conclude that the expression of NKG2C and/or activating KIRs are not prerequisites for the emergence of adaptive NK cells.

### CD2 and CD16 Synergistically Activate Adaptive NK Cells

**Figure 5:**
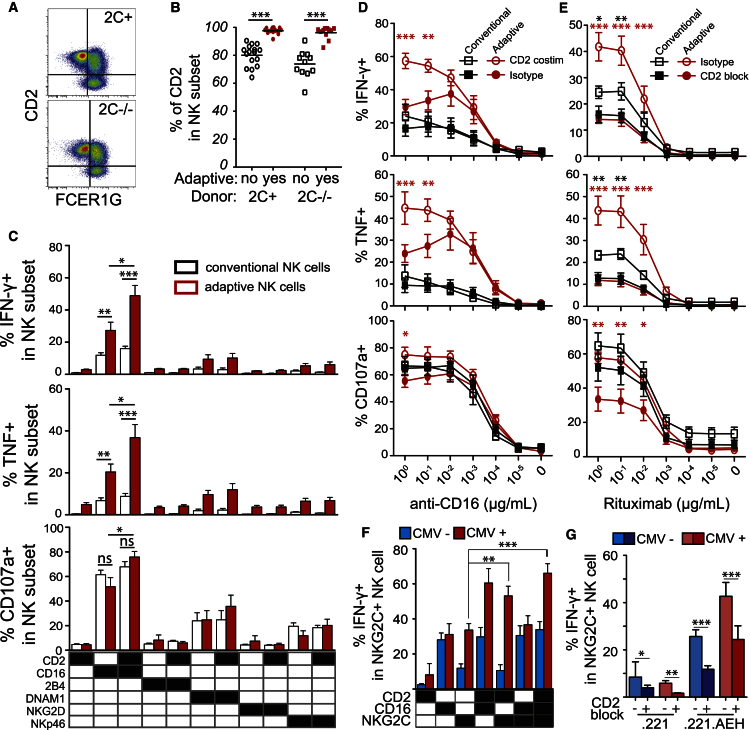
Synergistic Effect of CD2 and CD16 in Adaptive NK Cells (A and B) Representative FACS (A) plot and summary graph (B) of CD2 expression in adaptive NK cells of *NKG2C*^+^ and *NKG2C*^−/−^ donors. (C) Functional assay of conventional (white) CD56^dim^ NK cells as compared to adaptive (red) NK cells from *NKG2C*^−/−^ (n = 10) donors. Cell-surface expression of CD107a and intracellular expression of TNF and IFN-γ were assessed after 6 hr of redirected stimulation with P815 using mouse anti-human antibodies, as indicated (5 μg/ml). (D) Cell-surface expression of CD107a and intracellular expression of TNF and IFN-γ production in conventional (black lines) versus adaptive (red lines) NK cells of five NKG2C^−/−^ donors after 6 hr of redirected stimulation with P815 cells and anti-CD16 alone (closed symbols) or anti-CD2 (5 μg/ml) together with anti-CD16 (open symbols) at indicated concentrations. Significant differences of adaptive NK cell responses after CD16 stimulation compared to CD16^+^CD2 stimulation are depicted. (E) Cell-surface expression of CD107a and intracellular expression of TNF and IFN-γ production in conventional (black lines) versus adaptive (red lines) NK cells of five NKG2C^−/−^ donors after 6 hr of stimulation with RAJI cells coated with the indicated concentration of anti-CD20 (rituximab) in presence (close symbols) or absence (open symbols) of CD2 blocking. Significant differences of adaptive (red stars) and conventional (black stars) NK cell responses after rituximab stimulation with or without CD2 blocking are depicted. (C–E) Adaptive subsets in NKG2C^−/−^ donors were defined by the FcεR1γ^–^CD57^+^ phenotype. (F) Intracellular IFN-γ production in the NKG2C^+^ subset of CMV^+^ (red, n = 5) and CMV^–^ (blue, n = 5) donors after 6 hr of redirected stimulation with P815 and indicated combination of mouse anti-human CD2, CD16, and NKG2C antibodies (5 μg/ml each). (G) Intracellular IFN-γ production in the NKG2C^+^ subset of CMV^+^ (red, n = 8) and CMV^–^ (blue, n = 8) donors after 6 hr of stimulation with 721.221 cells expressing (221.AEH) or not (221) HLA-E. Error bars represent the SEM.

The finding that adaptive NK cell responses can occur independently of NKG2C and activating KIRs prompted us to revisit the potential role of other activating receptors expressed by NK cells. We found a profound downregulation of NKp46, stable expression or weak downregulation of 2B4, NTB-A, CRACC, and NKG2D and an increased expression of DNAM-1 and CD2 in adaptive NK cells of both *NKG2C*^−/−^ and *NKG2C*^+^ donors (Figures 5A, 5B, and S5A). Next, we addressed whether any particular activating receptors could co-stimulate human adaptive NK cells in a fashion similar to that described for DNAM-1 in Ly49H-driven responses in the mouse (Nabekura et al., 2014). Since it was recently shown that antibody-mediated recognition of CMV-infected cells can drive the expansion of adaptive NK cells (Lee et al., 2015), we tested the ability of CD2 (Figures 5C and S5B), 2B4, and DNAM-1 (Figure S5C) to co-stimulate the CD16 pathway in adaptive NK cells from both NKG2C^−/−^ and NKG2C^+^ donors. Agonistic stimulation of CD2 and CD16 using antibody-coated P815 cells revealed a striking synergistic interaction between CD2 and CD16 that was not observed for any other receptor combinations or in conventional NK cells. Thus, ligation of CD16 together with CD2 led to an increase in IFN-γ and TNF-producing cells compared to CD16 stimulation alone (Figures 5C and S5B). Titrating the dose of CD16 revealed that CD2 engagement enhanced the maximum response compared to CD16 crosslinking alone without influencing the response threshold (Figure 5D).

Next, we explored the potential contribution of CD2 to antibody-triggered responses by adaptive and conventional NK cells. To this end, we made use of the CD20^+^ RAJI B cell lymphoma line, which expresses CD58, the ligand of CD2. We monitored functional responses in the two subsets following incubation with anti-CD20 (rituximab)-coated RAJI cells. CD2 blockade led to a profound decrease of IFN-γ and TNF production as well as degranulation (Figure 5E). The costimulatory effect of CD2 was evident at low rituximab concentrations, suggesting that this pathway may boost the CD16 responses in the context of low levels of immunoglobulins. The blocking of CD2 also revealed a modest but significant synergy between CD2 and CD16 in conventional NK cells for IFN-γ and TNF production (Figure 5E). Notably, blockade of CD2 abrogated the difference in functional responses between adaptive and conventional NK cell subsets, suggesting that CD2 co-stimulation is a crucial element of the enhanced antibody-dependent responses by adaptive NK cells.

In *NKG2C*^+^ donors, NKG2C has a unique ability, alongside CD16, to trigger functional responses in resting NK cells without the need for additional co-activation (Luetke-Eversloh et al., 2014). Therefore, we tested the potential synergy between CD2, CD16, and NKG2C in the NKG2C-expressing subset from HCMV^–^ (conventional phenotype) and HCMV^+^ (adaptive phenotype) donors. Functional assays with agonistic monoclonal antibodies (mAbs) revealed synergies between NKG2C and CD2 in adaptive NK cells of HCMV^+^ individuals but not in conventional NK cells (Figure 5F). Blockade of CD2 interactions with its natural ligand in target cell assays with HLA-E-expressing 221 cells had a broader effect and diminished the response in both conventional and adaptive NK cells (Figure 5G), potentially attributed to an additional effect on target cell adhesion (Hahn and Bierer, 1993). Together these results reveal a unique role for CD2 in specifically boosting functional responses mediated through CD16 and NKG2C in adaptive NK cells.

### CD2 Co-stimulation Boosts the CD16 Signaling Cascade

**Figure 6:**
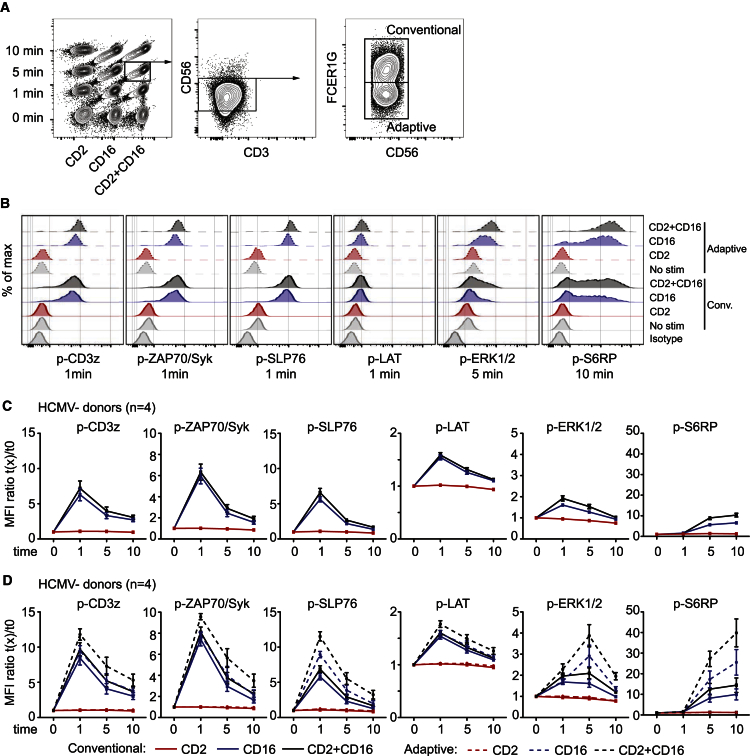
CD2 Co-activates the CD16-Induced Signaling Pathway in Adaptive NK Cells (A) Barcoding and gating strategy used to identify adaptive and conventional NK cells in the phosphoflow cytometry analysis. (B–D) Phosphorylation of the indicated phospho-epitopes in adaptive or conventional NK cells after CD2 and/or CD16 crosslinking. (B) Representative histogram plots at the indicated stimulation time point. (C and D) Mean fluorescence intensity (MFI) fold change of the indicated phospho-epitopes in conventional subset (solid line) of four HCMV^–^ (C) and the conventional (solid line) or adaptive (dotted line) subsets of four HCMV^+^ (D) individuals. Among the four HCMV^+^ donors, three were NKG2C^+^ and one was NKG2C^−/−^. Error bars represent the SEM.

To dissect the mechanism underlying CD2 costimulation in adaptive NK cells, we monitored the phosphorylation kinetics of key signaling molecules downstream of CD16, including CD3-ζ, ZAP70/Syk, SLP76, LAT, ERK1/2 (MAP kinase pathway), and S6RP (mTORC pathway), using phosphoflow cytometry (Figures 6A–6D) (Long et al., 2013). Unlike T cells (Kaizuka et al., 2009), no signaling events was induced in conventional or adaptive NK cells when triggered with anti-CD2 alone (Figures 6B–6D). In contrast, CD16 crosslinking induced phosphorylation of all signaling molecules tested in both NK subsets. In line with the functional readouts, CD16-induced phosphorylation of SLP76, ERK1/2, and S6RP was stronger in adaptive compared to conventional NK cells (Figure 6D). In conventional NK cells, we noted a weak synergy between CD2 and CD16 that was significant for the phosphorylation of ERK1/2 after 1 min (mean ratio 1.6 versus 1.9, p = 0.0006) and S6RP after 10 min (mean ratio 6.6 versus 10.3, p < 0.0001). In adaptive NK cells, however, co-ligation of CD2 and CD16 led to significantly higher levels of phosphorylation of all signaling molecules. This synergistic induction of signaling in adaptive NK cells was particularly pronounced for ERK1/2 after 5 min (mean ratio 2.9 versus 3.9, p = 0.017) and S6RP after 10 min (mean ratio 25.5 versus 39.8, p = 0.0066). Taken together, these results demonstrate that the costimulation of CD16 by CD2 is mechanistically linked to the synergistic induction of the MAP kinase and mTORC pathways.

## Discussion

During the last decade, it has become clear that NK cells possess the ability to calibrate their functional potential and respond to pathogenic challenges in a fashion that is commonly attributable to cells within the adaptive immune system. However, the natural drivers behind these responses remain largely unknown. In the mouse, the activating receptor Ly49H has probably evolved to counteract the decoy major histocompatibility complex (MHC) class I molecule in MCMV, m157, which allows the virus to escape NK cells expressing the inhibitory receptor Ly49C (Forbes et al., 2014, Pyzik et al., 2014). However, Ly49H is not found in all mouse strains suggesting that it is not required for survival in natura, despite the protection it confers against MCMV in certain laboratory strains (Arase et al., 2002). In the human, the activating NKG2C receptor is expressed by a majority of adaptive NK cells responding to HCMV. However, individuals lacking this receptor are perfectly healthy and appear fully capable of controlling HCMV infection. Thus, the immunological control of both mouse and human CMV must involve other activating NK cell receptors, compensatory immune responses by other lymphocyte subsets or a combination thereof. The comparison of NK cell repertoires in two large cohorts of healthy donors either lacking or expressing the *NKG2C* gene allowed us to address these possibilities in the human.

Here, adaptive NK cell responses in *NKG2C*^−/−^ individuals were limited to CMV-seropositive donors and occurred at similar frequencies as the previously described adaptive NK cell expansions in *NKG2C*^+^ donors. Although these expansions shared phenotypic, epigenetic, and functional attributes with NKG2C-expressing expansions, most of the identified NK cell populations lacked all known drivers of adaptive NK cell responses, including activating KIRs (Béziat et al., 2013, Della Chiesa et al., 2014). The question raised, then, is which other activating signals may be involved in driving adaptive NK cell responses in *NKG2C*^−/−^ donors. To address this, we first excluded the involvement of CD94/NKG2E by confirming that CD94 triggering and stimulation with HLA-E^+^ targets failed to activate NKG2C^−^ adaptive NK cells. This outcome is in accordance with the results of Orbelyan et al., showing that NKG2E is retained within the endoplasmic reticulum due to hydrophobic amino acids in the extracellular domain of the protein (Orbelyan et al., 2014).

A broad profiling of activating receptor expression on adaptive NK cells from *NKG2C*^−/−^ donors, we noted increased levels of DNAM-1 and CD2, two receptors that synergize with NKp46 to trigger resting NK cells (Bryceson et al., 2006). DNAM-1 expression is enhanced in educated NK cells and cooperates with LFA-1 to form stable target cell conjugates (Enqvist et al., 2015). In mice, DNAM-1 marks NK cell maturation (Martinet et al., 2015) and was found to be critical for the expansion of Ly49H^+^ NK cells after MCMV infection (Nabekura et al., 2014). Although our results do not exclude the involvement of DMAM-1 in the early phase of adaptive NK cell expansion and differentiation in the human, we did not observe costimulatory potential of this receptor in adaptive NK cells. Therefore, we turned our attention to CD2. CD2 is a major coactivating receptor expressed on NK and T cell subsets (Davis and van der Merwe, 1996); it recognizes CD58, a ligand expressed on a wide variety of tissues (Smith and Thomas, 1990). In resting conventional NK cells, cross-linking of CD2 with NKp46 increases the intracellular calcium flux, but not cytokine production or degranulation (Bryceson et al., 2006). Extending these results, we here describe a potent synergy between CD2 and CD16 that is unique to adaptive NK cells. Furthermore, CD2 ligation co-stimulated NKG2C-mediated responses in adaptive NK cells from *NKG2C*^+^ donors, suggesting that CD2 plays a broad role in the functionality of adaptive NK cells.

Notably, patients with homozygous missense mutations in CD16 have poor natural cytotoxicity, which is attributable to the loss of physical interactions between CD16 and CD2 (Grier et al., 2012). CD16 stabilizes the expression of CD2 and the two molecules co-localize at the immune synapse allowing CD2 to tap into the signaling pathway downstream of CD16. CD16 and NKG2C are associated with dimers of immunoreceptor tyrosine-based activation motif (ITAM)-containing molecules, FcεR1γ and/or CD3-ζ for CD16 and DAP12 for NKG2C (Lanier, 2008). CD3-ζ and DAP12 are expressed in all NK cells, while FcεR1γ is often downregulated in adaptive NK cells (Lee et al., 2015, Schlums et al., 2015, Zhang et al., 2013). As a consequence, in adaptive NK cells, CD16 is mostly associated with CD3-ζ/CD3-ζ homodimers (eight ITAMs) instead of CD3-ζ/FcεR1γ (six ITAMs) or FcεR1γ/FcεR1γ (four ITAMs) dimers. Since CD2 signaling was shown to be dependent on CD3-ζ (Moingeon et al., 1992, Vivier et al., 1991), it is tempting to speculate that this quantitative difference in ITAM motif contributes to the enhanced CD16 responses in adaptive NK cells. Supporting this notion, our data show that CD2 co-activation of CD16 in adaptive FcεR1γ^–^ NK cells leads to an increase phosphorylation of CD3-ζ as well as of all the other signaling molecules we tested. However, only a fraction of adaptive NK cells are FcεR1γ^–^ (Schlums et al., 2015), and NKG2C^+^FcεR1γ^+^ adaptive NK cells also exhibited heightened responses to CD2 and CD16 costimulation (data not shown), suggesting that alternative signaling pathways may be involved.

Adaptive NK cells displayed enhanced phosphorylation of ERK and S6RP upon CD16 and CD2 co-activation. ERK is a major hub of signal transduction in the MAP-kinase pathway, whereas S6RP is downstream of the mTORC1 complex; both are late signaling events involved in numerous central processes in lymphocytes, including proliferation, differentiation, and cytokine production (Dong et al., 2002, Pollizzi and Powell, 2014). Recently, mTORC pathway activity was shown to be essential for mouse NK cell development (Marçais et al., 2014). Our results suggest that this pathway should also be scrutinized for its potential role in the development of adaptive NK cell responses. The signaling cascade integrates different signals and functions as a signal amplifier. Therefore, an initially relatively small difference can get larger further down in the cascade. This fit with the gradually increasing differences observed in the present study: p-CD3z/p-ZAP70 < p-SLP76 < p-Erk. Indeed, we observed a tendency for increased phosphorylation of all proximal readouts following CD2 and CD16 costimulation of adaptive NK cells. Thus, although a major difference of phosphorylation can be excluded for these early signaling events, more subtle variations might remain unseen due to a lack of sensitivity.

Given the synergy observed between NKG2C and CD2, it is plausible that CD2 can also cooperate with other DAP12-coupled receptors such as activating KIRs, although this possibility was not specifically examined here. It will be of interest to explore whether CD2 costimulation of CD16-driven responses influence the functional reprogramming of the cell during expansion in a fashion similar to that noted in the context of T cell exhaustion in autoimmunity and infection (McKinney et al., 2015).

The expansion of adaptive NK cells in the absence of activating KIRs and NKG2C suggests that a combination of other NK receptors and or external factors contribute to elicit such responses. In this context, antibody-mediated recognition of viral antigens as a driver of adaptive NK cell responses is particularly appealing (Lee et al., 2015). Since CD2 is expressed at high levels on all adaptive NK cell subsets and significantly boost the response of NK cells to CD16 ligation, it is conceivable that recognition of CD58 on HCMV-infected cells play a role in shaping adaptive NK cell responses. HCMV has tropism for epithelial cells, endothelial cells, myeloid cells, and fibroblasts, all of which express CD58 (Revello and Gerna, 2010). CMV infection also causes loss of HLA class I (Tandon and Mocarski, 2012). Thus, CMV-infected cells fulfill all criteria for efficiently stimulating self-KIR^+^ adaptive NK cells via CD2 and CD16. Outstanding remaining questions are to define the cellular interactions that trigger the onset of adaptive NK cell responses as well as the cellular entities that maintain stable repertoires for many years during latency. Our results point to the necessity of looking beyond NKG2C and consider the possibility that broadly expressed receptors, such as CD2, provide essential co-stimulatory signals to NK cells, corresponding to signal 2 in the generation of adaptive T cell responses (Smith-Garvin et al., 2009).

Another major purpose of the current study was to examine whether the loss of NKG2C-driven responses had any influence on T cell-mediated immunity to HCMV. *NKG2C*^*−/−*^ donors displayed similar frequencies of CMV-specific T cells as the *NKG2C*^+^ donors, suggesting that lack of NKG2C had no major impact on the T cell response to the virus. However, we observed an accumulation of effector memory CD45RA^+^ CD8 T cells earlier in life in HCMV^+^*NKG2C*^−/−^ individuals, potentially indicating a stronger CD8 T cell response in the early phase of HCMV infection in the absence of NKG2C-driven adaptive NK cell immunity. This notion is supported by an extensive study of a rural population of Gambia, a country with almost universal HCMV seroprevalence; i.e., almost 100% of the population was HCMV^+^ by 6 years of age (Goodier et al., 2014). Although specific T cell immunity was not analyzed, immunoglobulin G (IgG) titers against HCMV were elevated in young individuals lacking the *NKG2C* gene. These results suggest that, despite a high level of redundancy within the NK cell compartment itself, the lack of *NKG2C* might also be partly compensated for by enhanced T and B cell responses, particularly during the early phases of HCMV infection. Possibly, an effective adaptive NK cell immunity helps to control the burden of HCMV infection before the emergence of efficient T and B cell immunity. Although adaptive NK cells displayed reduced degranulation responses, their enhanced ability to release cytokines in response to antibody-coated targets might help to fulfill this role and contribute to maintaining the virus silent during latency. The plasticity of adaptive NK cell responses in the absence of activating KIRs and NKG2C points to the importance of such responses within the innate immune system.

## Experimental Procedures

### Human Participants and Cells

This study was conducted in accordance with the Declaration of Helsinki and was approved by the ethics committee in Stockholm, Sweden. 2,208 random healthy blood donors were screened for NKG2C expression by flow cytometry. Donors lacking NKG2C expression were confirmed by PCR using the protocol described by Moraru et al. verifying homozygous deletion of *NKG2C* gene (Moraru et al., 2012a). 60 controls expressing NKG2C and 60 donors lacking the *NKG2C* gene were identified and enrolled in the study. For all donors, peripheral blood mononuclear cells (PBMCs) were cryopreserved for later use. Genomic DNA was isolated using the DNeasy Blood and Tissue Kit (QIAGEN).

### KIR and KIR-Ligand Typing and HCMV Serology

KIR ligands were determined using the KIR HLA ligand kit (Olerup SSP; QIAGEN) for detection of the HLA-Bw4, HLA-C1, and HLA-C2 motifs. KIR genotyping was performed by using quantitative KIR automated typing (qKAT) (Jiang et al., 2012). HCMV serology was determined using an ELISA-based assay on plasma obtained during sample preparation. Purified nuclear CMV antigen (AD 169) was used, and the cut-off level for seropositivity was an absorbance of ≥0.2 at a dilution of 1/100.

### Flow Cytometry

A list of fluorochrome-conjugated reagents used for stainings can be found in the Supplemental Experimental Procedures. Detailed protocols of flow cytometry staining, Stochastic neighbor embedding (SNE) analysis, functional flow cytometry assays, including T and NK cell functional assays and phospho flow cytometry experiments, are provided in the Supplemental Experimental Procedures. KIR repertoire analyses were performed according to the strategy previously described (Béziat et al., 2014).

### DNA Methylation Analysis

DNA methylation was analyzed as previously described (Luetke-Eversloh et al., 2014). The methylation levels of six CpG residues within the IFNG CNS1 region were analyzed via bisulfite conversion and pyrosequencing by Varionostic. Donors were selected based on the size of the three target subsets to ensure sufficient numbers of cells for methylation analysis after sorting.

### Statistics

For multiple group comparisons, one-way ANOVA, two-way ANOVA, or Kruskal Wallis nonparametric tests were applied. For single comparisons of independent groups, the Student’s t test or the Mann-Whitney test was performed. For single comparisons of matched groups, the paired Student’s t test or the Wilcoxon matched pairs test was performed depending on the sample size and distribution. For comparisons of qualitative variable, the Fisher’s exact t test was performed. In the relevant figures, n.s. indicates not significant; ^∗∗∗^p < 0.001; ^∗∗^p < 0.01; and ^∗^p < 0.05. Analyses were performed using GraphPad software.

## Author Contributions

L.L.L. performed experiments, analyzed data, and wrote the manuscript. J.L. performed and analyzed phospho-flow experiments. E.M.A. performed viSNE analysis. M.E., E.S., J.P.G., and V.Y.S.O. performed experiments. Q.H. performed epigenetic analysis. J.A.T. and J.J. performed KIR genetic analysis. S.L. provided crucial support for sample collection. M.S. performed HLA typing. K.T., H.G.L., C.R., and J.T. analyzed data and contributed to the writing of the manuscript. K.J.M. coordinated research efforts, supervised research work and data analysis, and wrote the manuscript. V.B. coordinated research efforts, performed experiments, supervised research work and data analysis, and wrote the manuscript.

## Supplemental Information

Document S1. Supplemental Experimental Procedures, Figures S1–S5, and Table S1

Document S2. Article plus Supplemental Information
