# Supplementary material for: Critical Role of CD2 Co-stimulation in Adaptive Natural Killer Cell Responses Revealed in NKG2C-Deficient Humans
Source: Cell Rep. 2016 Apr 21;15(5):1088–99. doi: 10.1016/j.celrep.2016.04.005 (PMC4858565; doi:10.1016/j.celrep.2016.04.005)
Supplement: Document S1. Supplemental Experimental Procedures, Figures S1–S5, and Table S1 [file mmc1.pdf]

Cell Reports, Volume *15*

Supplemental Information

Critical Role of CD2 Co-stimulation in Adaptive Natural Killer Cell Responses Revealed in NKG2C-Deficient Humans

Lisa L. Liu, Johannes Landskron, Eivind H. Ask, Monika Enqvist, Ebba Sohlberg, James A. Traherne, Quirin Hammer, Jodie P. Goodridge, Stella Larsson, Jyothi Jayaraman, Vincent Y.S. Oei, Marie Schaffer, Kjetil Taskén, Hans-Gustaf Ljunggren, Chiara Romagnani, John Trowsdale, Karl-Johan Malmberg, and Vivien Béziat

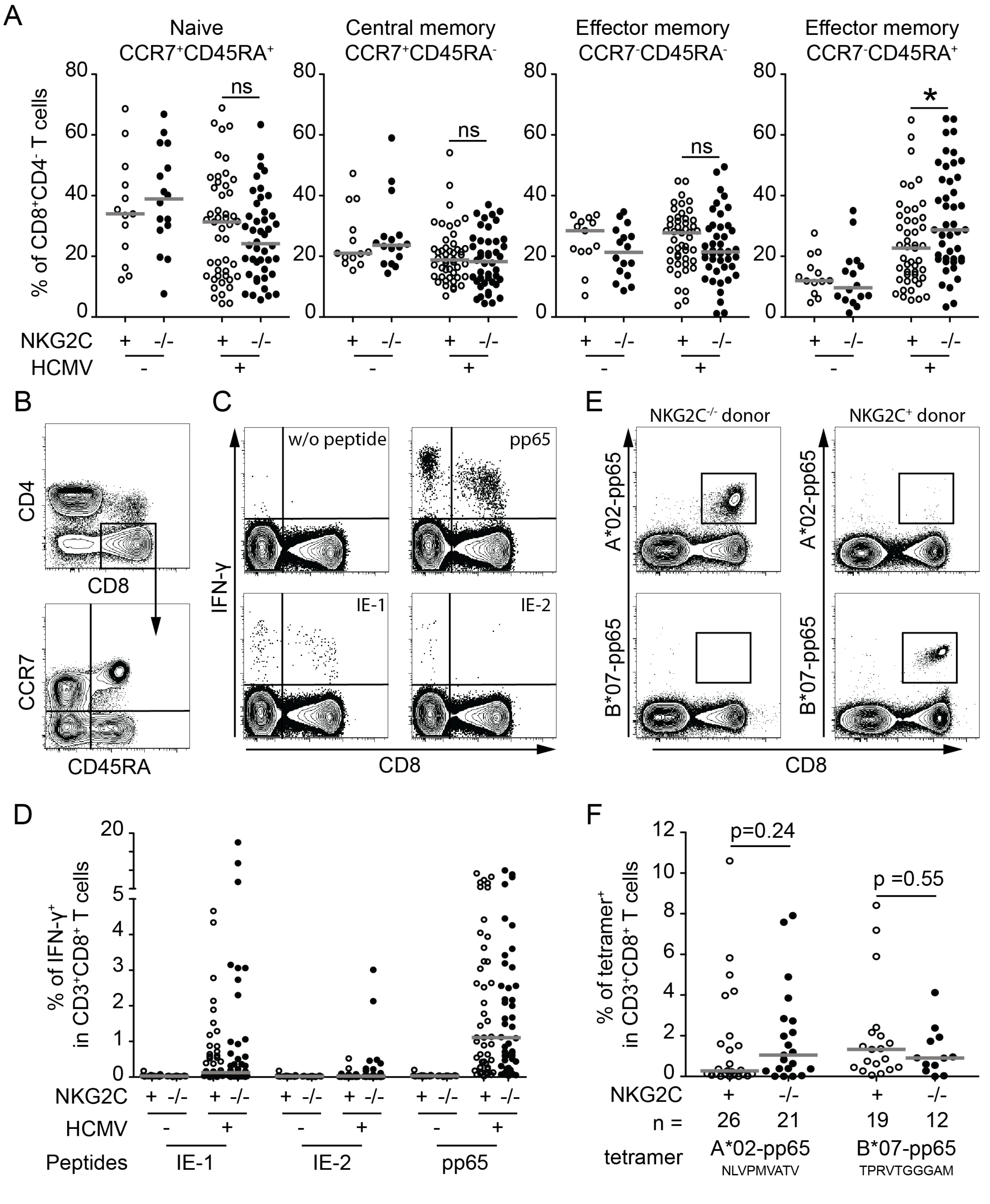

Liu *et al* Suppl. Fig. 1

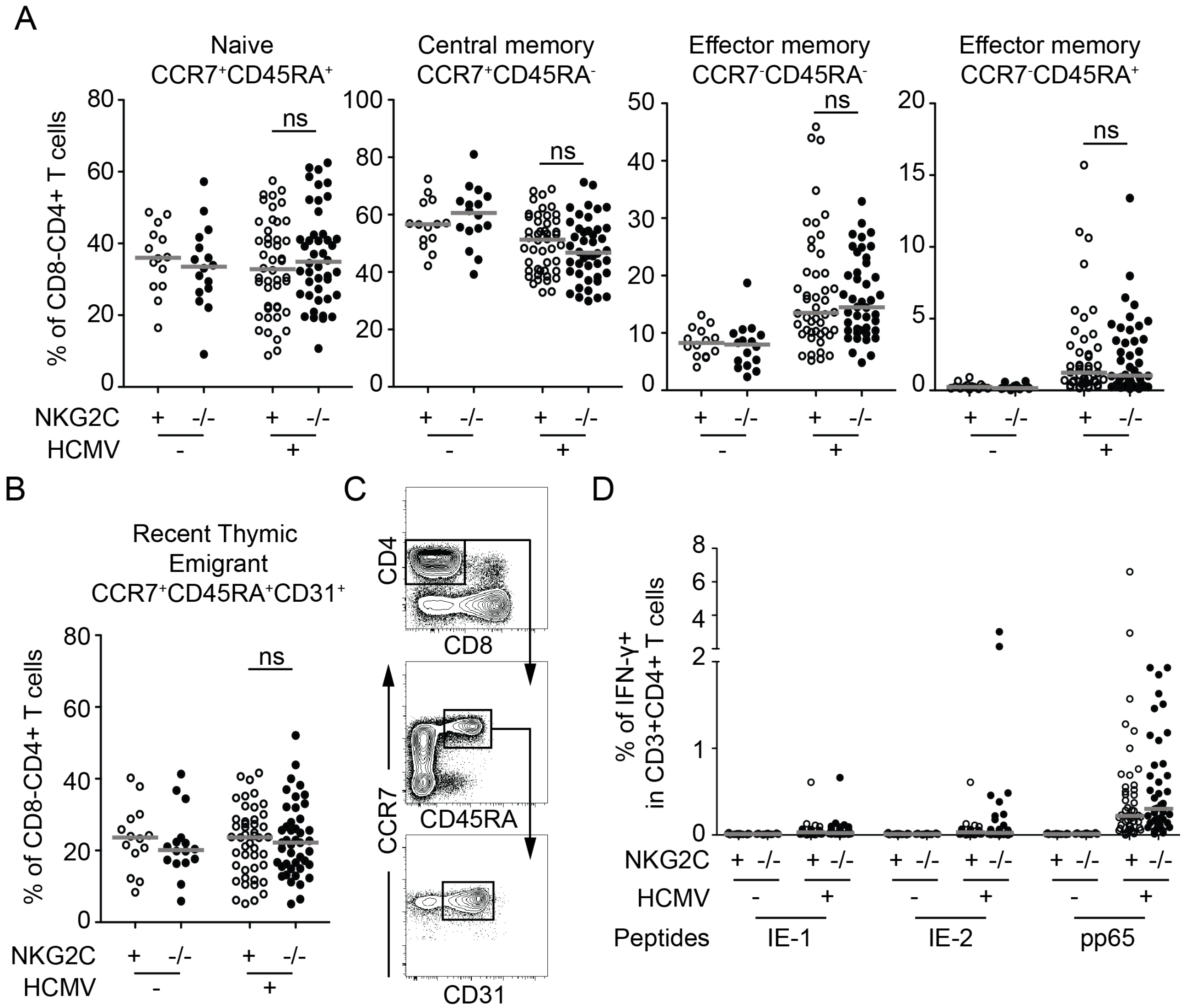
A
Naive
CCR7+CD45RA+
Central memory
CCR7+CD45RA-
Effector memory
CCR7-CD45RA-
Effector memory
CCR7-CD45RA+
% of CD8-CD4+ T cells
ns
NKG2C
HCMV
B
Recent Thymic
Emigrant
CCR7+CD45RA+CD31+
C
CD4
CD8
CCR7
CD45RA
CD31
D
% of IFN-γ+
in CD3+CD4+ T cells
Peptides
IE-1
IE-2
pp65

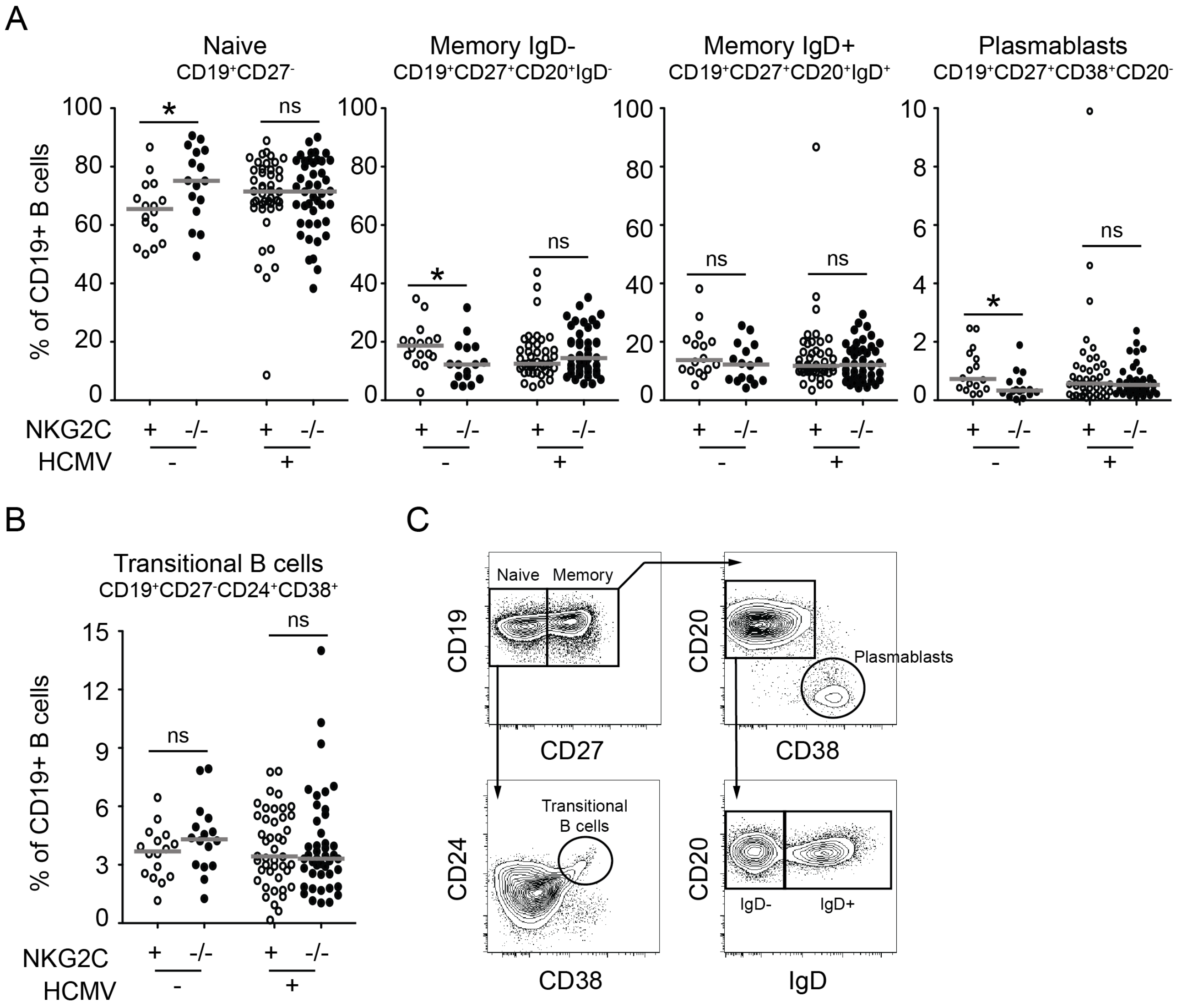

Liu *et al* Suppl. Fig. 3

A

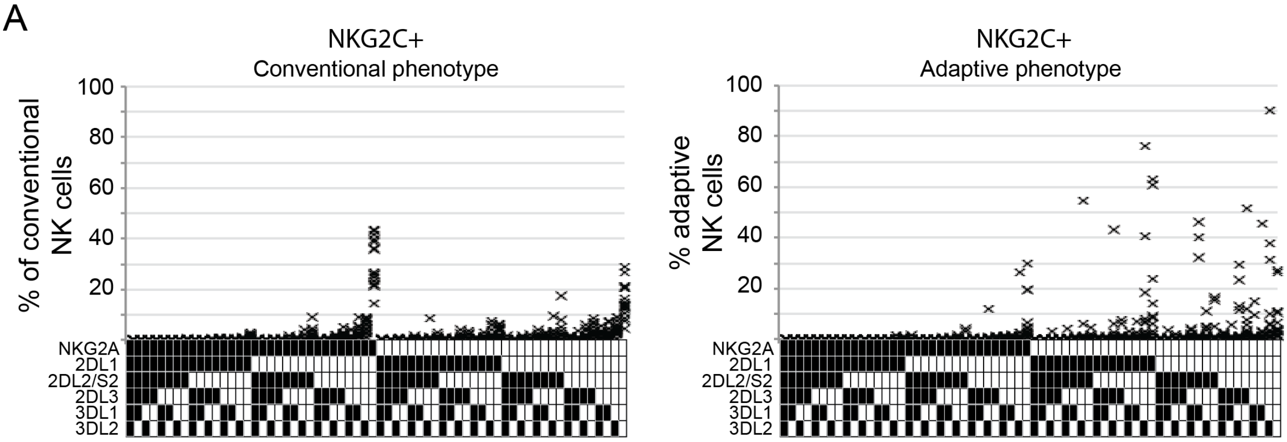

Liu *et al* Suppl. Fig. 4

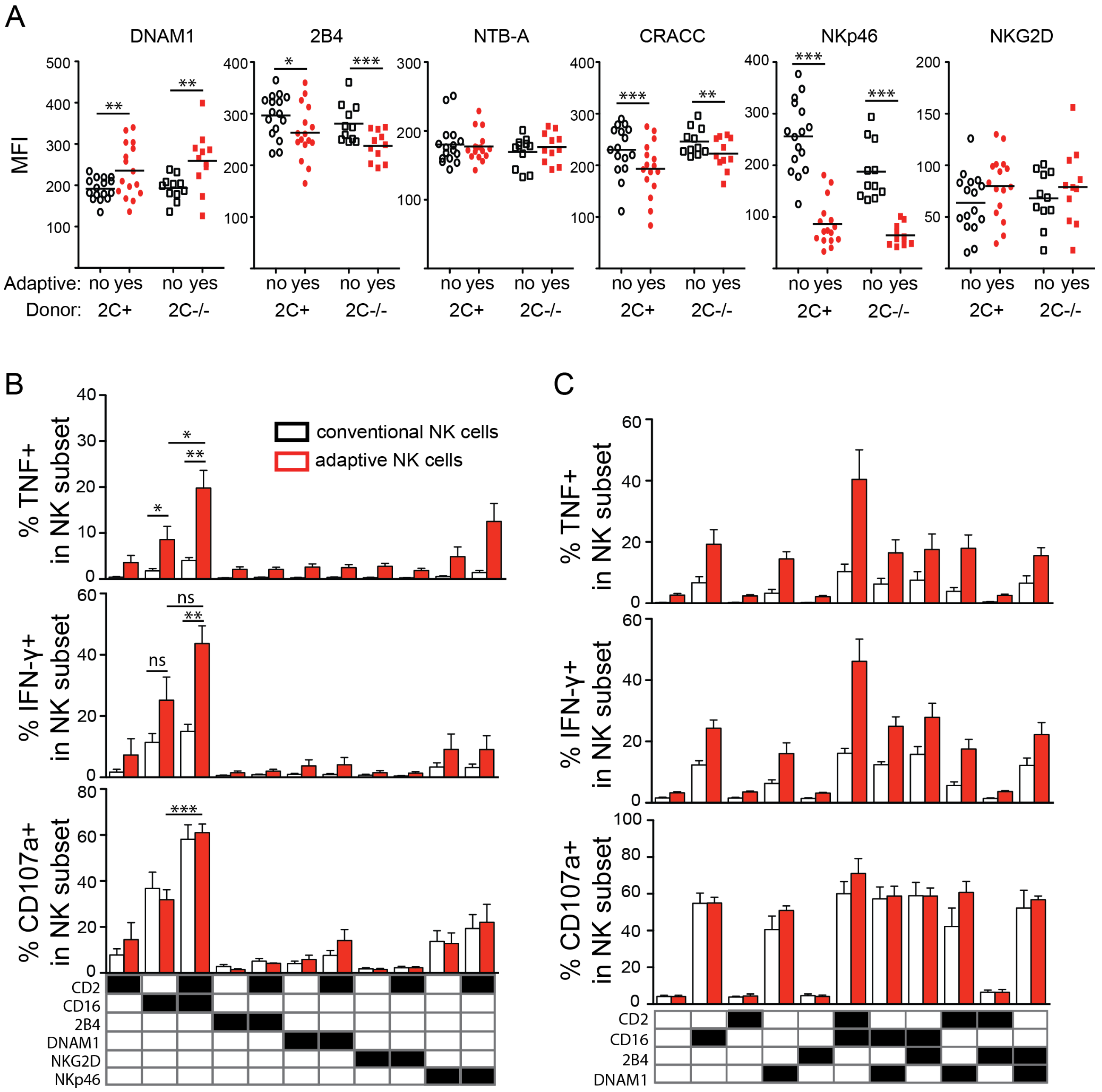

Liu *et al* Suppl. Fig. 5

Supplementary Figure 1. Phenotype and function of CD8 T cells in NKG2C$^{-/-}$ individuals (related to Figure 1). (A) Summary graphs of the frequency of CD8 T cell differentiation subsets in *NKG2C*$^{+}$ and *NKG2C*$^{-/-}$ donors stratified by their HCMV serology. (B) Gating strategy used to identify CD8 T cell differentiation subsets. (C-D) Representative FACS plot (C) and summary graph (D) of IFN-γ production by total CD8^{+} T cells of *NKG2C*$^{+}$ and *NKG2C*$^{-/-}$ donors after overnight stimulation with overlapping peptides of HCMV IE-1, IE-2 or pp65 proteins. Donors were stratified based on HCMV serology as indicated. (E-F) Representative FACS plot (E) and summary graph (F) of HCMV-specific CD8 T cells detected using HLA-A*02 and HLA-B*07 tetramers refolded with HCMV immunodominant epitopes, as indicated. Only HLA-A*02 and HLA-B*07 were analyzed. The grey lines represent the median value within each group.

Supplementary Figure 2. Phenotype and function of CD4 T cells in NKG2C$^{-/-}$ individuals (related to Figure 1). (A-B) Summary graphs of the frequency of CD4 T cell differentiation subsets in NKG2C^{+} and NKG2C$^{-/-}$ donors stratified by their HCMV serology. (C) Gating strategy used to identify CD4 T cell differentiation subsets. (D) Summary graph of IFN-γ production by total CD4^{+} T cells of NKG2C^{+} and NKG2C$^{-/-}$ donors after overnight stimulation with overlapping peptides of HCMV IE-1, IE-2 or pp65 proteins. Donors were stratified based on HCMV serology as indicated. Grey lines represent the median value within each group. Statistics: Mann-Whitney test.

Supplementary Figure 3. B cell phenotyping of *NKG2C*$^{-/-}$ individuals (related to Figure 1). (A-B) Frequency of various stages of B cell differentiation within the total CD19^{+} B cell of *NKG2C*$^{+}$ and *NKG2C*$^{-/-}$ donors stratified by their HCMV serology. (C) Gating strategy for Analysis of B cell differentiation. Grey lines represent the median value within each group. Statistics: Mann-Whitney test.

Supplementary Figure 4. NKG2A and KIR repertoires in NKG2C sufficient donors (related to Figure 3). Shown are NKG2A and KIR repertoires in conventional (left column) and adaptive (right column) NK cells of 17 *NKG2C*$^{+}$ donors.

Supplementary Figure 5. CD2 and DNAM-1 co-stimulation profile of adaptive NK cells (related to Figure 5). (A) Mean fluorescence intensity (MFI) of indicated activating receptors in adaptive and conventional NK cells from *NKG2C*$^{+}$ and *NKG2C*$^{-/-}$ donors (B) Functional profile of NKG2C-expressing adaptive NK cells (red bars) compared to conventional NK cells (white bars) in 5 *NKG2C*$^{+}$ donors after stimulation with the indicated agonistic mouse-antihuman antibodies. (C) Co-stimulation profiles in *NKG2C*$^{-/-}$ (n=5) donors of conventional (white bars) and adaptive (red bars) NK cells stimulated with the indicated agonistic mouse-antihuman antibodies. Cell surface expression of CD107a and intracellular expression of TNF and IFN-γ were assessed after 6 hours of redirected stimulation with P815 coated with mouse anti-human antibodies (5μg/ml).

Supplementary Table 1. Characteristics of NKG2C-/- and NKG2C+ donor cohorts (related to Figure 1)

	$NKG2C^{-/-}$ cohort (n=60)		$NKG2C^{+}$ cohort (n=60)	
HCMV serolology	$HCMV^{+}$ n=44 (73.3%)	$HCMV^{-}$ n=16 (26.7%)	$HCMV^{+}$ n=47 (78.3%)	$HCMV^{-}$ n=13 (21.7%)
Males, n (%)	20 (45.4%)	12 (75.0%)	16 (34.0%)	7 (53.8%)
Age, median (range)	50.0 (21-68)	43.0 (23-70)	51 (21-69)	43 (23-63)
HLA-A*02, n (%)	21 (47.7%)	12 (75.0%)	26 (55.3%)	9 (69.2%)
HLA-B*07, n (%)	12 (27.3%)	9 (56.3%)	19 (40.4%)	0 (0.0%)
Haplotype A/A, n (%)	13 (29.5%)	4 (25.0%)	11 (23.4%)	3 (23.1%)
HLA-C1/C1, n (%)	22 (50.0%)	6 (37.5%)	23 (48.9%)	6 (46.1%)
HLA-C2/C2, n (%)	8 (18.2%)	2 (12.5%)	3 (6.4%)	4 (30.8%)
HLA-C1/C2, n (%)	14 (31.8%)	8 (50.0%)	21 (44.7%)	3 (23.1%)
HLA-Bw4, n (%)	32 (72.7%)	12 (75.0%)	27 (57.5%)	9 (69.2%)

Supplemental Experimental Procedures

Antibodies and tetramers

Stainings were performed using a panel of the following antibodies (clone names are given in brackets): CD14-AF700 (HCD14), CD14-V500 (M5E2), CD19-PE.Cy5 (J3-119), CD19-V500 (HIB19), CD8-BV785 (RPA-T8), CD4-Qdot705 (S3.5), CD45RA-ECD (2H4LDH11LDB9), CD3-PE-Cy5.5 and PE-Cy5 (UCHT1), CCR7-PE-Cy7 and BV421 (G043H7), CD57-PB (HCD57), CD31-PE (WM-59), CD27-APC-Cy7 (0323), CD24-APC (eBioSN3), CD38-APC-Cy7 (HIT2), CD20-FITC (2H7), IgD-PE (IA6-2), CD57 purified (TB01), anti-mouse-IgM-EF650 (II/41), FCER1γ-FITC (rabbit polyclonal), CD7-PE-Cy7 (8H8.1), LILRB1/ILT2-PE (HP-F1), CD161-BV605 (HP-3G10), CRACC-PE (235614), NKG2A-APC or APC.AF750 or PE-Cy7 (Z199), NKG2C-PE or A488 (FAB138P), NTB-A-PE (292811) DNAM-1-PE-vio770 (Dx11), NKG2D-BV711 (1D11), CD16-AF700 (3G8), 2B4-PE (C1.7), NKp46-BV786 (9E2), CD2-PB (TS1/8), KIR2DL3-FITC (180701), KIR2DL1-APC (143211), KIR3DL1-AF700 (DX9), KIR2DS4-QD585 (179315), KIR3DL2-biotin (Dx31), KIR2DL2/L3/S2-PE-Cy5.5 (GL183), KIR2DL1/S1-PE-Cy7 (EB6). Dead cells were labeled with live/dead aqua (Life Technologies). Biotin-conjugated antibodies were visualized by using streptavidin-Qdot 585 or 605 (Life Technologies). Tetramers staining were performed using iTAG tetramer HLA-A*0201-PE (CMV pp65 NLVPMVATV) and iTAG tetramer HLA-B*0702-PE (CMV TPRVTGGGAM). After extracellular staining, cells were fixed and permeabilized by using a fixation/permeabilization kit (eBioscience) prior to intracellular staining. Samples were acquired using an LSR Fortessa 18-color flow cytometer (Becton Dickinson) and data were analyzed with FlowJo software version 9 (TreeStar). The BD LSR Fortessa instrument was equipped with a 100 mW 405 nm laser, a 100 mW 488 nm laser, a 50 mW 561 nm laser, and a 40 mW 639 nm laser.

T cell functional assay

Freshly thawed PBMCs were cultivated in complete medium (RPMI, glutamine, 10% FCS) at a final concentration of $5x10^6$ cells/mL in a 96-well U-bottom plate. Cells were stimulated, or not, with CMV^{pp65}, CMV^{IE1} or CMV^{IE2} overlapping peptides (JPT Technology, 1µg/mL for each peptide) in the presence of brefeldin A (GolgiPlug, BD Biosciences, 1/1000 final concentration). After 16 hours of incubation (37°C, 5% CO_2), the cells were stained for extracellular receptors, permeabilized (Fixation/permeabilization buffer, eBioscience), stained for intracellular IFN-γ-AF700 (B27) and analyzed by flow cytometry.

NK cell functional assay

Thawed PBMCs were rested overnight in complete medium and distributed at a final concentration of $2.5x10^6$ cells/mL in 96-well U-bottom plates. All target cells were added to PBMCs at a final concentration of $2.5x10^5$ cells/ml. For conventional functional assays, K562 or 221.AEH cells were used as target cells. For ADCC assays, RAJI cells were used as targets in the presence of 1µg/mL rituximab or the indicated concentration together with anti-CD2 (RPA-2.10, 5µg/ml) when indicated. For redirected functional assays, P815 murine mastocytoma cells were the targets. Anti-CD16 (3G8), anti-CD2 (RPA-2.10), anti-DNAM-1 (DX11), anti-2B4 (eBioC1.7), anti-Nkp46 (9E2), anti-NKG2D (1D11), anti-CD94 (131412) or anti-NKG2C-PE (134591) were added alone or in combinations in the indicated wells at a final concentration of 5µg/ml, unless otherwise specified (*e.g.*, CD16 titration). The cells were incubated for 6 hours (37°C, 5% CO_2) after the addition of monensin (GolgiStop, BD Biosciences, 1/1500 final concentration), brefeldin A (GolgiPlug, BD Biosciences, 1/1000 final concentration) and CD107a-BV421 (H4A3, 1/100 final concentration). After incubation, cells were washed and stained for extracellular receptors, permeabilized (Fixation/permeabilization buffer, eBioscience) and stained for intracellular TNF-APC (MAb11) and IFNγ-AF700 (B27) prior to analysis by flow cytometry.

For the cytokine stimulation assays, freshly thawed PBMCs (10^6 cells) were incubated 16 hours at 37°C and 5% CO_2 with 10ng/mL IL-12 and 100 ng/mL IL-18 in U-bottomed 96-well plates. After the incubation, cells were washed and stained for extracellular receptors, permeabilized (Fixation/permeabilization buffer, eBioscience) and stained for intracellular IFNγ-AF700 (B27) prior to analysis by flow cytometry.

Phospho flow cytometry

Thawed PBMCs were rested in complete medium for 3-5 hours and stimulated directly or rested overnight and subjected to NK cell-negative selection (NK cell isolation kit, Miltenyi) prior to stimulation, as described earlier (Kalland et al., 2011). To avoid variability due to sample processing, a fluorescent cell barcoding was applied, allowing simultaneous analysis of three stimulatory conditions at four different time points (Fig.7A). In brief, cells were incubated at 37°C in complete medium in suspensions between 6-50 M/mL for 10 min. Then, biotinylated CD2 (eBioscience, clone RPA-2.10) and / or biotinylated CD16 (Biolegend, clone 3G8) were added to final concentrations of 5 μg/mL each. After 1 min, the aliquot for the 0 min (unstimulated) sample was removed and mixed with Fix Buffer I (BD Biosciences). After one additional minute, stimulation was started by crosslinking the biotinylated antibodies via 50 μg/mL avidin (Thermo Fischer Scientific) and the aliquots for the 1 min, 5 min and 10 min samples were transferred into Fix Buffer I (BD bioscience) at the corresponding time points. Cells were fixed at 37°C for 10 min, washed and re-suspended in PBS. To allow combination of the differently stimulated samples into one pool, two dimensional fluorescent cell barcoding (FCB) was utilized. Samples were stained in distinct concentrations of amine-reactive pacific blue succinimidyl ester (Thermo Fisher Scientific) for the time points (0 min – 0.69 ng/mL, 1 min – 6.25 ng/mL, 5 min – 25 ng/mL and 10 min – 100 ng/mL) in combination with amine-reactive pacific orange succinimidyl ester (Thermo Fisher Scientific) for the different stimulations (CD2 – 10 ng/mL, CD16 –

100 ng/mL and CD2+CD16 – 500 ng/mL). After 20 min at RT, samples were washed twice in wash solution (PBS supplemented with 1% FCS and 0.09% sodium azide), combined, permeabilized (Perm Buffer III, BD Biosciences) and stored at -80°C. For thawing, samples were placed 20 min on ice. They were then washed in wash solution and stained with anti-CD3 PerCP-Cy5.5 (BD Biosciences, clone UCHT1), anti-CD56 ECD (Beckman Coulter, clone N901) and anti-FcεRIγ FITC or Alexa Fluor 488 (Merk Millipore, polyclonal) in combination with Alexa Fluor 647-conjugated phospho epitope-specific antibodies against CD3ζ (pY142), LAT (pY171), SLP76 (pY128), ZAP70/syk (pY319/pY352) (BD Bioscience), Erk1/2 (pT202/pY204), S6-ribosomal protein (S6RP, pS235/236) (Cell Signaling Technology) or isotype control IgG1κ (BD Biosciences) for 30 min at RT. After washing data were acquired on an LSR Fortessa (BD Biosciences) and analyzed with FlowJo v10.

Stochastic neighbor embedding (SNE) analysis

FCS files from all donors were imported into FlowJo version 9 (TreeStar) and NK cells were identified based on CD3 and CD56 expression. These events were exported for further processing using R version 3.1.0. with which 5000 events were randomly sampled from each file and then pooled. Two-dimensional Barnes-Hut t-distributed SNE was then performed with the Rtsne R package (http://CRAN.R-project.org/package=Rtsne). For the panel of differentiation markers, the SNE calculation was based on the parameters FCER1γ, LILRB1, CD7, NKG2C, CD161, CD56, pan-KIR2D and NKG2A, giving the best resolution of the highly differentiated adaptive NK cell subset in NKG2C$^{+/+}$ donors. SNE density plots (Figures 2A) were created using FlowJo and processed using Adobe Photoshop CS6. All other SNE plots were generated using the ggplot2 R package (http://ggplot2.org/).
